# Relative Validity of a Short Food Frequency Questionnaire for Disadvantaged Families in Hong Kong

**DOI:** 10.3390/nu15122668

**Published:** 2023-06-08

**Authors:** Vicky Wai-Ki Chan, Crystal Ying Chan, Becky Pek-Kei Hoi, Joyce Ho-Yi Chan, Liz Li, Maggie Ying-Yee Li, Edwin Shun-Kit Chung, Henry Ho-Fai Sin, Eliza Lai-Yi Wong, Kenneth Ka-Hei Lo

**Affiliations:** 1 Department of Food Science and Nutrition, The Hong Kong Polytechnic University, Hong Kong SAR, China; vickycwk.chan@polyu.edu.hk (V.W.-K.C.); sin-liz.li@polyu.edu.hk (L.L.); 2 JC School of Public Health and Primary Care, The Chinese University of Hong Kong, Hong Kong SAR, China; ychan@cuhk.edu.hk (C.Y.C.); beckyhoi@cuhk.edu.hk (B.P.-K.H.); hoyijoycechan@cuhk.edu.hk (J.H.-Y.C.); maggieyyli@cuhk.edu.hk (M.Y.-Y.L.); shunkitchung@cuhk.edu.hk (E.S.-K.C.); henrysin@cuhk.edu.hk (H.H.-F.S.); 3 Center for Health Systems and Policy Research, JC School of Public Health and Primary Care, The Chinese University of Hong Kong, Hong Kong SAR, China; 4 Research Institute for Smart Ageing, The Hong Kong Polytechnic University, Hong Kong SAR, China

**Keywords:** food frequency questionnaire, dietary assessment, relative validity, Hong Kong, lower-income families

## Abstract

Individuals with lower socioeconomic status are more vulnerable in securing good nutritional quality. It was also found that people who had received a lower education level had greater difficulty in completing the conventional dietary assessment such as a food frequency questionnaire (FFQ). Previous studies have demonstrated the validity of a short FFQ in Hong Kong’s pregnant women, but its validity among a wider community was still unknown. For the present study, we aimed to validate a short FFQ among disadvantaged communities in Hong Kong. Amongst 103 individuals participating in a dietary intervention programme, their dietary data were collected by FFQs and three-day dietary records. Relative validity was assessed by correlation analysis, cross-tabulation, one-sample *t*-test, and linear regression. In general, water and total energy intake had significant correlations (0.77 for crude water intake and 0.87 for crude total energy intake) between values reported by FFQ and dietary records, good agreement (both with over 50% of observations falling into the same quartile), and insignificant differences between assessment methods reported by one-sample *t*-test and linear regression. Meanwhile, several nutrients had good agreement in terms of the values reported by FFQ and dietary records, such as energy from total fat, carbohydrates, total fat, cholesterol, phosphorus, and potassium. The results of this study demonstrated that the short version FFQ could be a convenient assessment tool of multiple dietary behaviors, especially in total energy and water intakes.

## 1. Introduction

Since Coronavirus disease 2019 (COVID-19) was declared a pandemic on 11 March 2020 [1], it has been a huge public health burden worldwide with multiple waves of outbreaks, including in Hong Kong [2]. Despite the effectiveness in controlling the spread of the virus in the earlier waves of outbreaks [3], the pandemic, temporary lockdowns, and fear of contracting the infection have generally increased public anxiety [4]. Meanwhile, the prolonged period of social distancing measures raised concerns about maintaining a healthy diet [5]. During the first phase of the COVID-19 pandemic in Hong Kong (May to June 2020), a telephone survey was conducted with 724 adults to measure their changes in lifestyle habits during a period in which social distancing measures were being imposed [5]. In general, the frequency of eating fruit and vegetables significantly increased (both *p* < 0.05), which might relate to the higher awareness of maintaining a healthy diet and improving immunity. However, individuals with a lower socioeconomic status were more likely to have fewer healthier eating behaviors. Socioeconomic status (SES) indicates one’s position in society, and it is classified by income, education, and occupation. In 2022, around 1.5 million Hong Kong people (20% of the total population) lived below the poverty line. During the COVID-19 pandemic, low-income families had doubled their food expenditure and almost half of them were unemployed. They became more vulnerable to household food insecurity due to financial constraints [6]. According to previous studies, people with a lower monthly household income dined out more frequently, and consumed less fruit and vegetables (all *p* < 0.05) in their diet during the pandemic period. In addition, individuals without a full-time or part-time job had a larger increase in sugary drinks consumption (*p* < 0.01) compared to those who remained employed [5]. It was also suggested that, compared with higher socioeconomic groups, Chinese people from lower socioeconomic classes had a higher prevalence of diabetes [7,8]. These results indicated the vulnerability of individuals with lower socioeconomic status in securing good nutritional quality, particularly during the pandemic. Hence, it is important to understand the nutritional needs of disadvantaged families in formulating health promotion strategies.

A Food Frequency Questionnaire (FFQ) is a common, valid, and reliable instrument for dietary assessment, which examines the usual intake of a wide range of nutrients. However, completing FFQ is usually a lengthy process, often including over 100 food items [9]. It was observed that education level would affect one’s ability to report the amount of food intake, where people who had received a lower education level had greater difficulty in administering the FFQ, leading to recall bias [10]. That demonstrates the need to validate a brief and culturally specific version FFQ for disadvantaged communities, which will help them to estimate their dietary intake with less recalling burden.

In a previous study, a short version FFQ was developed for 45 pregnant women from Hong Kong, which demonstrated good reliability and validity in reflecting dietary intake of various nutrients and food groups, especially for fruits and fibre intake [11]. However, its validity in assessing the diet of a wider population has not been examined. Therefore, the purpose of the present study is to assess the diet of Hong Kong’s families with lower socio-economic conditions with the newly developed short FFQ, and evaluate its relative validity as a dietary assessment tool for disadvantaged communities.

## 2. Materials and Methods

### 2.1. Subjects and Study Design

All participants were recruited from a community intervention project to investigate the effect of a 6-month lay health worker intervention in diabetes management among the key caregivers living in sub-divided flats units in Hong Kong [12]. Participants were recruited by four non-profit organizations (NPOs) from both the Kwai Tsing and Kowloon City districts. Participants who were: (1) in charge of food preparation for the family, (2) ethnically Chinese and aged at or above 18 but less than 65, (3) with ≥ 1 risk factor(s) or symptoms for type II diabetes mellitus according to the Hong Kong Reference Framework for Diabetes Care for Adults in Primary Care Settings [13], (4) holders of a Hong Kong identity card, and (5) able to speak and understand Chinese were eligible for this study. Participants who were cognitively incompetent, not able to give consent, and with co-living family members already participating in this study were excluded.

Eligible participants were given a study information sheet and an ethics consent form, which were explained in person by a trained research assistant before providing the written informed consent. The protocol was approved by the Joint Chinese University of Hong Kong—New Territories East Cluster Clinical Research Ethics Committee of the Chinese University of Hong Kong (2021.313) and was registered in the Chinese Clinical Trial Registry (ChiCTR2100052080).

### 2.2. Data Collection

All participant information was self-reported with the aid of an electronic survey form. Collected information was encrypted and only research staff involved could have access. The following demographic characteristics were collected: sex, age, body mass index (BMI), education level, working status, and total monthly family income. After obtaining consent, the trained research staff would interview the participants to complete the FFQ and three-day dietary records before they entered the intervention programme.

### 2.3. Dietary Assessments

#### 2.3.1. Short Version FFQ

The 50-item short version FFQ was developed from a validated 266-item FFQ being used among Hong Kong adults by an expert group of dietitians, nutritionists, and nurses with face validity [14]. In this short version FFQ, foods with similar nutrient composition were combined into one category by averaging the nutrient data of all included items, which were then used for computing participants’ mean consumption of specific nutrients. For the grouping criteria, nutrients of interest and major characteristics of food were of major concern. The grouping details are shown in Table S1. To facilitate the recall of dietary intake, related food categories were allocated adjacently in the FFQ.

Since the present study is nested within a community interventional trial, each participant only completed one FFQ through phone interview, and, therefore, the reproducibility of the FFQ was not evaluated. For each food item, participants were required to report the consumed portion size in pieces, cups, tablespoons, teaspoons, millilitres, or grams, as well as the frequency of consumption as per day, week, or month, referring to the photo of standardized eating utensils and food photo booklet provided beforehand.

#### 2.3.2. Three-Day Dietary Records

Three-day dietary records, one on a weekend day and two on weekdays, were collected before administrating the FFQ, acting as a reference in evaluating the validity of the FFQ. The dietary record forms and a food photo illustrating the size of a bowl and tablespoon were provided through smartphone messages to participants a week before conducting the FFQ interview. Instructions on reporting dietary consumption in the previous 24 h, including all food and beverages consumed, were given by trained research staff. A detailed example was also provided to participants as a reference, including portion sizes, ingredients, and brand names. As with the FFQ, pieces, cups, tablespoons, teaspoons, millilitres, or grams were used to present the consumed portion sizes.

#### 2.3.3. Dietary Data Analysis

All dietary data collected from both the FFQ and three-day dietary records were computed using the ESHA Research Food Processor SQL: Nutrition Analysis and Fitness Program (Copyright 2022, ESHA Research).

#### 2.3.4. Prevalence of Deficient or Excessive Dietary Intake

Dietary data collected from three-day dietary records were used to calculate the prevalence of deficient or excessive dietary intakes of the 103 participants. A total of 20 parameters were included, namely total energy, energy from total fat, energy from saturated fat, energy from trans-fat, protein, carbohydrates, total dietary fibre, total sugar, cholesterol, water, vitamin C, calcium, copper, iron, magnesium, manganese, phosphorus, potassium, sodium, and zinc. For most parameters, the Chinese Dietary Reference Intakes (DRIs) established by the Chinese Nutrition Society were used as reference values [15], except for sodium, cholesterol, and energy from trans-fat, which referred to the recommended nutrient intakes as suggested by the World Health Organization (WHO) [16].

### 2.4. Statistical Analysis

For parametric tests, continuous variables were presented as the mean (standard deviations). For qualitative variables, number (percentage) was presented. Pearson correlation coefficients (r) were used to evaluate the relative validity of FFQs with respect to the average of three-day dietary records. With the residual method developed by Willett [17], nutrient intakes were energy adjusted as an alternative analysis, and the Pearson correlation after energy adjustment was computed. A one-sample *t*-test was then utilized to examine whether the differences in nutrient intake between the FFQ and the average of three-day dietary records were statistically significant.

In addition, with the use of linear regression, the associations between the differences in nutrient intake and the average nutrient intakes of the two assessment methods were modelled. The associations would be insignificant if the discrepancy between the FFQ and dietary records did not increase with the increment of dietary intake.

To measure the agreement between the FFQ and the average of three-day dietary records, the crude estimates and energy-adjusted nutrient intakes were both classified using quartile analysis. The percentage of agreement was then computed to evaluate how well the two methods could categorize individuals consistently (i.e., equivalent or adjacent quartiles (±1 quartile)). Bland–Altman plots were used to visualize the differences in the FFQ and dietary records’ reported values of total energy intake and three common macronutrients, namely carbohydrates, protein, and total fat. All statistical analyses were two-tailed and the level of significance was set to *p*-value less than 0.05, using the statistical package SPSS version 24.0 ( IBM Corp., Armonk, NY, USA).

## 3. Results

### 3.1. Demographic Characteristics

A total of 103 participants (96.1% female) completed the FFQ and three-day dietary records and were included in the analysis. Demographic characteristics at enrolment are presented in Table 1. The mean age of participants was 42.1 years old and mean BMI was 25.6 kg/m^2^. Among 103 participants, 80.6% of them were housewives or were unemployed, and 61.2% received junior secondary education or below. Some 41.8% of their total family income was below HKD 10,000 while 39.8% of them were making HKD 10,000–20,000 per month as a family.

### 3.2. Prevalence of Excessive or Deficient Dietary Intake

Dietary data of three-day dietary records collected from 103 participants were used to calculate the prevalence of an excessive or deficient intake of 20 nutrients, which is shown in Table 2. In general, a majority of participants had an excessive intake of sodium (91.3%) and energy from total fat (68.9%). Furthermore, they had a deficient intake of total energy (82.5%), total dietary fibre (97.1%), water (77.7%), vitamin C (62.1%), calcium (92.2%), iron (92.2%), magnesium (89.3%), manganese (95.2%), and potassium (66.0%). The mean daily intake of energy and selected nutrients of the short version FFQ and dietary records of 103 participants are shown in Table 3.

### 3.3. Relative Validity of FFQ and Three-Day Dietary Records

#### 3.3.1. Correlation Analysis

The correlation between the FFQ and three-day dietary records is shown in Table 4. Significant positive correlations (r ≥ 0.7) were found in 10 variables, namely total energy, total fat, water, magnesium, phosphorus, potassium, energy from total fat, protein, carbohydrates, and total dietary fibre. After energy adjustment, water remained a consistently strong correlation (r = 0.75) and most variables still demonstrated a good correlation (0.5 ≤ r ≤ 0.7), except for total fat (r = 0.49) and phosphorus (r = 0.40).

#### 3.3.2. Cross-Tabulation of Dietary Data Reported by FFQ and Dietary Records

Cross-tabulation was adopted to evaluate the agreement of dietary intakes between the FFQ and three-day dietary records. The results are displayed in Table 5. For crude mean intakes, the percentage of agreement within the same quartile varied from 37.9% (iron) to 71.8% (total energy). In general, around 87% of crude intakes estimated by the FFQ and three-day dietary records were within the same or adjacent quartiles. After energy adjustment, substantial changes in the percentage of agreement within the same quartile were observed in the following 7 variables: total fat (from 58.3% to 41.7%), saturated fat (from 50.5% to 32.0%), trans fat (from 43.7% to 31.1%), copper (from 42.7% to 29.1%), magnesium (from 49.5% to 37.9%), phosphorus (from 53.4% to 38.8%), and potassium (from 56.3% to 41.7%). In general, the percentage of agreement within the same or adjacent quartiles was 80.9% after adjusting for energy.

#### 3.3.3. One-Sample *t*-Test and Linear Regression for the FFQ and Dietary Records

In Table 6, a one-sample *t*-test was used to determine whether the differences between the values reported by the FFQ and three-day dietary records were significant (*p*-value ≤ 0.05). Insignificant differences were found in the following six variables: total energy, cholesterol, protein, water, carbohydrates, and potassium. In addition, linear regression was applied to determine whether the differences in intakes collected by the FFQ, and the three-day dietary records increased significantly with the levels of dietary intakes (*p*-value ≤ 0.05). Insignificant differences were found in the following 14 variables: total energy, total energy from total fat, total sugar, total fat, cholesterol, water, vitamin C, calcium, copper, iron, magnesium, manganese, phosphorus, and zinc.

#### 3.3.4. Bland–Altman Plots for Different Nutrients

**Figure 1:**
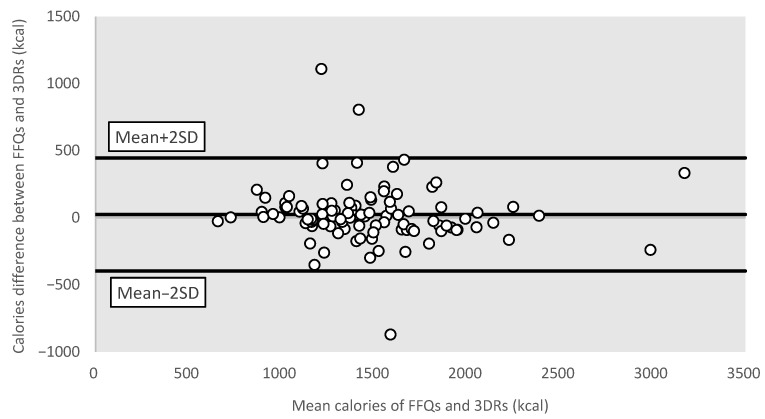
Bland–Altman plot of total energy. Abbreviations: FFQ: Food Frequency Questionnaire; SD: standard deviation; 3DRs: three-day dietary records.

**Figure 2:**
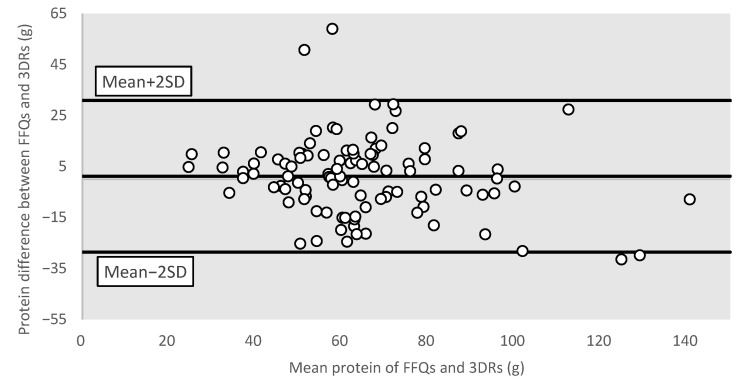
Bland–Altman plot of protein. Abbreviations: FFQ: Food Frequency Questionnaire; SD: standard deviation; 3DRs: three-day dietary records.

**Figure 3:**
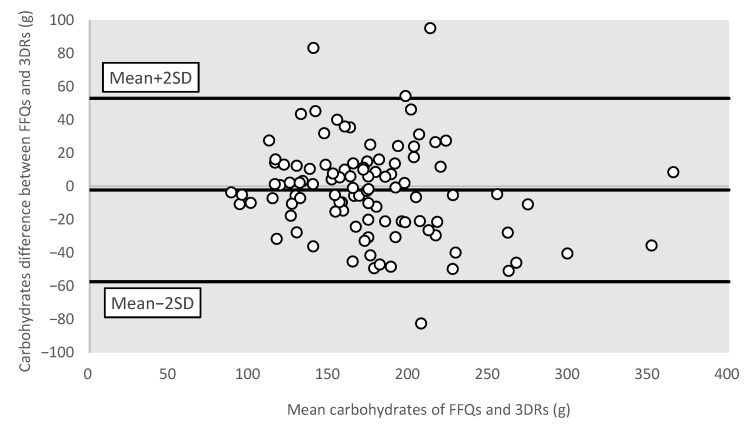
Bland–Altman plot of carbohydrates. Abbreviations: FFQ: Food Frequency Questionnaire; SD: standard deviation; 3DRs: three-day dietary records.

**Figure 4:**
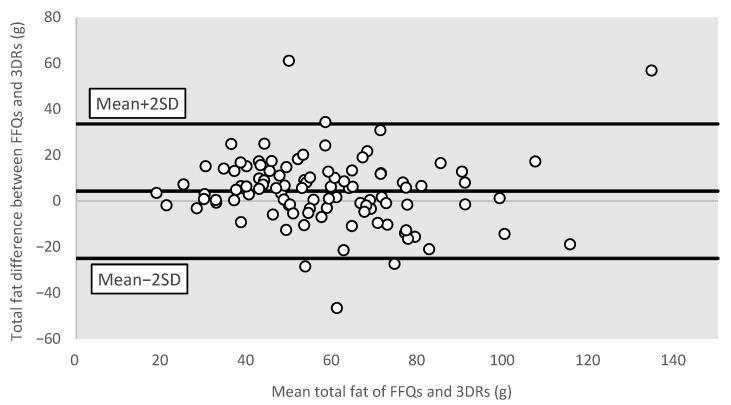
Bland–Altman plot of total fat. Abbreviations: FFQ: Food Frequency Questionnaire; SD: standard deviation; 3DRs: three-day dietary records.

Bland–Altman plots were used to further visualize the measurement differences in total energy (Figure 1), protein (Figure 2), carbohydrates (Figure 3), and total fat (Figure 4) between the FFQ and three-day dietary records. A total of 3 outliers were found in both Figure 1 and Figure 3. In Figure 2, 4 outliers were identified, and 5 outliers were found in Figure 4.

## 4. Discussion

### 4.1. Summary of Main Findings

The present study has examined the validity of a short version FFQ to measure the dietary intake of 103 participants from low-income families in Hong Kong, and their prevalence of excessive or deficient nutrient intakes was also examined. In general, the majority of participants had excessive intakes of sodium and energy from total fat, while having a deficient intake of total energy, total dietary fibre, water, vitamin C, calcium, iron, magnesium, manganese, and potassium. After examining the relative validity by correlation analysis, cross-tabulation of dietary data collected by the FFQ and three-day dietary records, one-sample *t*-tests, and linear regressions, water and total energy intake have shown good agreement in all analyses; while energy from total fat, energy from saturated fat, carbohydrates, total dietary fibre, total fat, saturated fat, phosphorus; and potassium have good agreement in terms of the values reported by the FFQ and dietary records.

### 4.2. Prevalence of Deficient or Excessive Dietary Intakes

With reference to data collected from dietary records, the prevalence of dietary intakes not meeting the Chinese DRI or WHO recommendations have been computed. In general, around 70% and 91% of participants had excessive intakes of energy from total fat and sodium, respectively. This indicated that participants were having a diet with a high fat and sodium intake. Their mean intake of sodium was 3334.8 mg per day, which was much higher than the upper limit of sodium intake (2000 mg per day) recommended by the WHO [18], and was similar to the average sodium intake (3520 mg per day) of Hong Kong’s adult population [19]. As suggested by the Department of Health, high frequencies of eating-out and consumption of preserved food potentially contributed to a higher sodium intake of Hong Kong’s population [19]. High sodium intake could inhibit renal expression of vascular endothelial growth factor and its receptor, inducing the elevation of blood pressure [20]. Therefore, having a high-sodium diet in the long term can associate with a higher risk of hypertension. On the other hand, over 90% of the participants were not having a sufficient intake of total dietary fibre and calcium. The suggested dietary fibre intake for Chinese adults ranged from 25 g to 30 g per day [21]. However, the mean dietary fibre intake of participants was 12.2 g per day. Low dietary fibre intake could lead to increased risk of cardiovascular diseases as soluble dietary fibre is responsible for inducing excretion of bile and lowering blood cholesterol levels. In addition, insufficient dietary fibre intake increased the risk of constipation as dietary fibre may increase the size of fecal and stimulating peristalsis [22]. For Chinese adults, the suggested calcium intake was 650 mg per day for those aged under 50, and 800 mg per day for those aged above 50 [21]. The mean calcium intake of participants was as low as 449.6 mg per day. According to the first Hong Kong Total Diet Study conducted by the Centre for Food Safety, the calcium intake of over 90% of adults in Hong Kong is under the recommended dietary intake and one of the possible reasons was that, in general, Hong Kong’s people had a lower consumption of dairy products [23]. Without sufficient dietary calcium intake, the calcium concentration in plasma would decrease; boosting the release of parathyroid hormone (PTH) and induced bone resorption; hence increasing the risk of osteoporosis due to low bone mineral density [24]. Apart from dietary fibre and calcium, the majority of participants also had insufficient intake of vitamin C, water, iron, magnesium, manganese, potassium, and total energy, which reflected that their dietary behaviors were not desirable. Despite a relatively small sample size, the present analysis has provided a glimpse of the nutritional needs of Hong Kong’s disadvantaged communities, especially in the midst of COVID-19.

### 4.3. Relative Validity of Short Version FFQ

Pearson correlation coefficients ranging from 0.5 to 0.7 reflected a good correlation while coefficients larger than 0.7 showed a very strong correlation between the two assessment methods [25]. From our results, the correlations between the two assessment methods were good to very strong for most parameters, except for iron, manganese, and sodium, for which only acceptable correlations were observed. According to the previous reviews, people from lower socioeconomic classes had more frequent consumption of processed foods, which included a high content of sodium in general [26]. Due to inflated food prices during the COVID-19 pandemic, especially for meat and vegetables, individuals from disadvantaged communities had limited choices of food so it was difficult to maintain a consistent diet, and this might lead to the fluctuating dietary values reported by the FFQ and three-day dietary records.

When comparing to the three-day dietary records, the short version FFQ has overestimated the intake of vitamin C, calcium, and energy from trans-fat while underestimating the intake of sodium. This might be because we attempted to combine food items into one category to shorten the FFQ but limited the ability to distinguish intake of micronutrients. For example, in the fruit category, fruits such as currants or lemons with a high vitamin C content were combined with fruits such as pears and peaches, which had a relatively lower vitamin C content. This is an example of the trade-off between identifying nutritional needs in the community effectively and conducting a comprehensive dietary assessment with accurate reporting of all included nutrients.

The agreement between the two assessment methods was evaluated by cross-classification. Reported values for most parameters were within the same or adjacent quartiles and insignificant differences between assessment methods were reported by one-sample *t*-test and linear regression. In addition, the one-sample *t*-test was adopted to evaluate the significance of differences in values reported by the two assessment methods. Insignificant differences were found in six variables, namely total energy, protein, carbohydrate, cholesterol, water, and potassium, which was additional support for the agreement in reported values.

From our findings, after considering all the statistical approaches for validation, the short version FFQ was particularly useful in distinguishing participants’ intake of water and total energy; which had significant correlation (0.77 for crude water intake and 0.87 for crude total energy intake) between values reported by the FFQ and dietary records; good agreement (both over 50% of observations falling into same quartile), and insignificant differences between assessment methods reported by the one-sample *t*-test and linear regression were observed for these two variables. Apart from the above variables, the short FFQ also demonstrated accuracy in capturing intakes of energy from total fat, total fat; and phosphorus (with significant correlations of 0.76, 0.76, and 0.75, respectively, good agreement, and insignificant differences between assessment methods reported by linear regression); carbohydrates; potassium (with significant correlations of 0.85 and 0.75, respectively, good agreement, and insignificant differences between assessment methods reported by one-sample *t*-test); and cholesterol (with a significant correlation of 0.62 and insignificant differences between assessment methods reported by one-sample *t*-test and linear regression).

### 4.4. Limitations

Although the short version FFQ has demonstrated good agreement in measuring multiple nutrient intakes of people from Hong Kong with lower socio-economic status, there were a few limitations of our study. Firstly, this study was conducted during COVID-19 so we could only provide photos of standardized eating utensils for participants to estimate portion size, and communicate with them through phone calls. This may have led to misreporting of consumption portions. Secondly, the short version FFQ did not differentiate the meat category into high-fat or low-fat, which led to a data discrepancy in measuring energy from fat. Third, since this study was nested within an intervention study, reproducibility of the FFQ was not evaluated. Since the majority of the participants were mainly middle-aged women, the results might not be generalized to Hong Kong’s population. Lastly, inter-rater and test-retest reliability were not examined as limited by the study design (nested within an intervention study). However, our findings have suggested the potential of using short version FFQs to identify nutritional needs in nutrient level for disadvantaged communities.

## 5. Conclusions

The results of this validation study demonstrated that the present short version FFQ could be a convenient assessment tool for multiple dietary behaviors, especially for total energy intake and water. However, it has overestimated the intake of vitamin C, calcium, and energy from trans-fat while underestimating the intake of sodium, which may be a trade-off between the accuracy and convenience of data collection. When developing short FFQs in further studies, the accuracy of micronutrient and fat intakes should also be considered.

## Figures and Tables

**Table 1:** Characteristics of 103 participants.

Characteristics	Mean (±SD)/N (%)
Age (Years)	42.1 (±8.0)
Sex (Female)	99 (96.1)
BMI at enrolment (kg/m^2^)	25.6 (±1.8)
Education	
Junior Secondary School or less	63 (61.2)
Senior Secondary School	30 (29.1)
Tertiary or above	10 (9.7)
Working Status	
Working	20 (19.4)
Non-working #	83 (80.6)
Total Family Income (HKD)	
<10,000	43 (41.8)
10,000–20,000	41 (39.8)
>20,000	19 (18.5)

Abbreviations: body mass index (BMI); standard deviation (SD). # The unemployed and housewives were both included.

**Table 2:** Prevalence of excessive or deficient dietary intake.

Parameters	Prevalence (%)
Above DRIs	
Energy from Total Fat ^b^	68.9%
Energy from Saturated Fat ^b^	37.9%
Energy from Trans Fat ^d^	1.9%
Total Sugar ^b^	38.8%
Cholesterol ^d^	45.6%
Sodium ^d^	91.3%
Below DRIs	
Total Energy ^a^	82.5%
Protein ^e^	26.2%
Carbohydrates ^e^	13.6%
Total Dietary Fibre ^c^	97.1%
Water ^c^	77.7%
Vitamin C ^e^	62.1%
Calcium ^e^	92.2%
Copper ^e^	14.6%
Iron ^e^	92.2%
Magnesium ^e^	89.3%
Manganese ^c^	95.2%
Phosphorus ^e^	17.5%
Potassium ^c^	66.0%
Zinc ^e^	52.4%

Abbreviation: DRIs: Dietary Reference Intakes. ^a^ Prevalence based on Estimated Energy Requirement (EER). ^b^ Prevalence based on Acceptable Macronutrient Distribution Range (AMDR). ^c^ Prevalence based on Adequate Intake (AI). ^d^ Prevalence based on World Health Organization (WHO) recommended daily intake. ^e^ Prevalence based on Estimated Average Requirement (EAR).

**Table 3:** Mean daily intake of energy and selected nutrients of the short version FFQ and three-day dietary records (*n* = 103).

Parameters	FFQ					Three-Day DRs				
	Mean	SD	Median	25th	75th	Mean	SD	Median	25th	75th
Energy (kcal)	1486.2	410.2	1422.9	1205.3	1700.0	1462.1	427.8	1405.7	1204.2	1694.3
Energy from Total Fat (kcal)	539.1	189.7	517.5	424.9	636.8	500.9	197.1	496.5	347.6	629.9
Energy from Saturated Fat (kcal)	149.9	52.0	139.8	115.4	175.4	136.2	63.9	120.2	87.8	179.3
Energy from Trans Fat (kcal)	5.2	2.6	4.6	3.3	6.3	1.8	1.8	1.2	0.7	2.2
Protein (g)	65.4	20.3	62.2	50.0	76.8	64.2	23.2	60.6	49.0	73.8
Carbohydrates (g)	174.4	47.6	169.4	146.3	198.1	176.6	53.3	171.5	135.4	202.8
Total Dietary Fibre (g)	12.9	4.2	12.3	10.3	15.0	12.2	5.0	10.9	8.2	16.0
Total sugar (g)	46.1	19.9	44.1	30.8	59.8	35.8	19.0	33.8	21.4	44.8
Total fat (g)	60.1	21.1	57.9	47.3	70.8	55.8	21.9	55.2	38.7	70.6
Saturated fat (g)	16.7	5.8	15.5	12.8	19.5	15.1	7.1	13.4	9.8	19.9
Trans fat (g)	0.6	0.3	0.5	0.4	0.7	0.2	0.2	0.1	0.1	0.2
Cholesterol (mg)	316.6	134.0	289.7	218.6	380.2	307.0	143.9	282.9	205.6	406.0
Water (g)	2156.8	779.1	2070.6	1514.4	2705.9	2084.8	708.5	1989.1	1579.1	2668.6
Vitamin C (mg)	96.5	41.7	86.8	66.3	128.3	77.1	46.5	71.6	42.2	101.8
Calcium (mg)	517.4	203.6	462.1	365.8	623.2	449.6	212.1	413.4	315.3	532.5
Copper (mg)	1.2	0.4	1.1	0.9	1.3	0.9	0.4	0.9	0.6	1.1
Iron (mg)	9.8	3.1	9.5	7.5	11.5	8.2	3.5	7.3	5.8	9.9
Magnesium (mg)	227.0	68.3	217.7	183.5	257.6	200.3	70.8	193.6	153.7	227.3
Manganese (mg)	2.8	1.2	2.6	2.0	3.4	2.5	1.1	2.3	1.9	3.1
Phosphorus (mg)	872.6	273.7	826.7	672.9	995.0	818.0	288.0	776.3	643.2	947.0
Potassium (mg)	1856.4	549.0	1763.8	1475.7	2175.3	1837.6	652.5	1735.6	1371.8	2146.9
Sodium (mg)	2989.2	711.3	2955.7	2610.0	3343.5	3334.8	1212.2	3121.6	2451.8	4147.2
Zinc (mg)	8.0	2.7	7.7	6.3	9.8	6.6	2.8	6.1	4.8	7.7

Abbreviations: DR: Dietary Record; DRIs: Dietary Reference Intakes; EAR: Estimated Average Requirement; FFQ: Food Frequency Questionnaire; g: gram; kcal: kilocalorie; mg: milligram; SD: Standard Deviation.

**Table 4:** Pearson correlation coefficient between the FFQ and the average of three-day dietary records (*n* = 103).

Parameters	Pearson Correlation Coefficient	
	Crude	Energy Adjusted
Energy (kcal)	0.87 **	-
Energy from Total Fat (kcal)	0.76 **	-
Energy from Saturated Fat (kcal)	0.68 **	-
Energy from Trans Fat (kcal)	0.61 **	-
Protein (g)	0.76 **	0.50 **
Carbohydrates (g)	0.85 **	0.68 **
Total Dietary Fibre (g)	0.78 **	0.69 **
Total sugar (g)	0.68 **	0.62 **
Total fat (g)	0.76 **	0.49 **
Saturated fat (g)	0.68 **	0.44 **
Trans fat (g)	0.61 **	0.49 **
Cholesterol (mg)	0.62 **	0.47 **
Water (g)	0.77 **	0.75 **
Vitamin C (mg)	0.60 **	0.55 **
Calcium (mg)	0.56 **	0.43 **
Copper (mg)	0.51 **	0.34 **
Iron (mg)	0.47 **	0.30 **
Magnesium (mg)	0.74 **	0.53 **
Manganese (mg)	0.49 **	0.27 **
Phosphorus (mg)	0.75 **	0.40 **
Potassium (mg)	0.75 **	0.61 **
Sodium (mg)	0.49 **	0.20 **
Zinc (mg)	0.56 **	0.21 **

Abbreviations: g: gram; kcal: kilocalorie; mg: milligram. ** indicates the level of significance at 0.01 level.

**Table 5:** Cross-classification of energy and nutrient intake quartiles from the FFQ and the average of three-day dietary records (*n* = 103).

Parameters	Crude ^a^			Adjusted for Energy ^a^		
	Same Quartile	Adjacent Quartile ^b^	Extreme Quartile ^c^	Same Quartile	Adjacent Quartile ^b^	Extreme Quartile ^c^
Energy (kcal)	71.8	24.3	3.9	-	-	-
Energy from Total Fat (kcal)	60.2	35.0	4.9	-	-	-
Energy from Saturated Fat (kcal)	50.5	37.9	11.7	-	-	-
Energy from Trans Fat (kcal)	46.6	37.9	15.5	-	-	-
Protein (g)	49.5	35.9	14.6	41.7	40.8	17.5
Carbohydrates (g)	61.2	30.1	8.7	61.2	30.1	8.7
Total Dietary Fibre (g)	52.4	39.8	7.8	46.6	42.7	10.7
Total sugar (g)	49.5	38.8	11.7	45.6	40.8	13.6
Total fat (g)	58.3	35.9	5.8	41.7	43.7	14.6
Saturated fat (g)	50.5	38.8	10.7	32.0	42.7	25.2
Trans fat (g)	43.7	41.7	14.6	31.1	37.9	31.1
Cholesterol (mg)	48.5	37.9	13.6	43.7	34.0	22.3
Water (g)	58.3	33.0	8.7	61.2	31.1	7.8
Vitamin C (mg)	49.5	39.8	10.7	45.6	37.9	16.5
Calcium (mg)	43.7	38.8	17.5	35.9	47.6	16.5
Copper (mg)	42.7	32.0	25.2	29.1	51.5	19.4
Iron (mg)	37.9	39.8	22.3	34.0	33.0	33.0
Magnesium (mg)	49.5	41.7	8.7	37.9	47.6	14.6
Manganese (mg)	43.7	39.8	16.5	38.8	39.8	21.4
Phosphorus (mg)	53.4	31.1	15.5	38.8	40.8	20.4
Potassium (mg)	56.3	35.0	8.7	41.7	38.8	19.4
Sodium (mg)	41.7	37.9	20.4	33.0	42.7	24.3
Zinc (mg)	45.6	35.9	18.4	39.8	35.0	25.2

Abbreviations: g: gram; kcal: kilocalorie; mg: milligram. ^a^ Percentage of all categories may not be rounded up to 100% exactly. ^b^ Same ± 1 quartile. ^c^ Same ± 2 quartiles.

**Table 6:** Results from one-sample *t*-test and linear regression.

Parameters	Mean Difference	*p*-Values	Beta-Coefficient	*p*-Values
Energy (kcal)	24.094	0.258	−0.045	0.395
Energy from Total Fat (kcal)	38.171	0.005 *	−0.043	0.557
Energy from Saturated Fat (kcal)	13.684	0.005 *	−0.243	0.006 *
Energy from Trans Fat (kcal)	3.387	<0.001 *	0.443	<0.001 *
Protein (g)	1.178	0.433	−0.151	0.040 *
Carbohydrates (g)	−2.221	0.425	−0.122	0.033 *
Total Dietary Fibre (g)	0.714	0.024 *	−0.211	0.003 *
Total sugar (g)	10.322	<0.001 *	0.051	0.561
Total fat (g)	4.298	0.004 *	−0.042	0.573
Saturated fat (g)	1.520	0.005 *	−0.243	0.006 *
Trans fat (g)	0.376	<0.001 *	0.443	<0.001 *
Cholesterol (mg)	9.628	0.423	−0.088	0.364
Water (g)	71.966	0.153	0.107	0.136
Vitamin C (mg)	19.358	<0.001 *	−0.136	0.178
Calcium (mg)	67.761	<0.001 *	−0.053	0.620
Copper (mg)	0.239	<0.001 *	−0.004	0.971
Iron (mg)	1.671	<0.001 *	−0.183	0.126
Magnesium (mg)	26.718	<0.001 *	−0.041	0.595
Manganese (mg)	0.335	0.003 *	0.100	0.389
Phosphorus (mg)	54.544	0.007 *	−0.058	0.444
Potassium (mg)	18.774	0.665	−0.197	0.010 *
Sodium (mg)	−345.572	0.001 *	−0.684	<0.001 *
Zinc (mg)	1.366	<0.001 *	−0.077	0.466

Abbreviations: DR: Dietary Recall; FFQ: Food Frequency Questionnaire; g: gram; kcal: kilocalorie; mg: milligram; SD: Standard Deviation. * indicates significant findings (*p*-value ≤ 0.05).

## Data Availability

The data presented in this study are available on request from the corresponding authors. The data are not publicly available due to privacy or ethical restrictions.

## Supplementary Materials

The following supporting information can be downloaded at: https://www.mdpi.com/article/10.3390/nu15122668/s1, Table S1: Comparison of food items included in the shortened and original version of FFQs.
