# Supplementary material for: Relative Validity of a Short Food Frequency Questionnaire for Disadvantaged Families in Hong Kong
Source: Nutrients. 2023 Jun 8;15(12):2668. doi: 10.3390/nu15122668 (PMC10304536; doi:10.3390/nu15122668)
Supplement: Supplementary file 1 [file nutrients-15-02668-s001.zip › nutrients-2345216-supplementary.pdf]

Table S1. Comparison of food items included in the shortened and original version of FFQs.

Food items from short FFQ	Food items from original FFQ
1. Refined grains, cooked	Rice
	Soft Rice
	Congee
	Noodles/Udon
	Instant Noodles
	Rice Noodles
	Macaroni
	Pasta
2. Whole grains, cooked	Whole grains, cooked
3. Refined bread	Chinese Steam Buns (Mann-Tau)
	Plain Rolls
	White Breads
	Sweet Rolls
4. Whole grain bread	Whole Wheat Breads
5. Refined cereals, dry	Oatmeal
	Corn Flakes
	Frosties
6. Whole grain cereals, dry	Whole grain cereals, dry
7. Fresh fruits	Oranges
	Grapefruits
	Apples
	Pears
	Bananas
	Honeydew Melons
	Watermelon
	Pineapples
	Strawberries
	Peaches
	Mangos
	Persimmons
	Kiwi fruits
	Apricots
	Prunes
	Grapes
	Lychee

	Longans
	Cherries
	Papayas
	Pomelo
8. Dried fruits	Dried Apricot
	Dried Prunes
	Raisins
	Dried Dates
9. Vegetables/Squash/Root/Stem	Choy Sum
	Bok Choy
	Chinese Kale
	Broccoli
	Cauliflowers
	Chinese Spinach
	Watercress
	Spinach
	Water Spinach
	Pea Shoots
	Cabbages
	Celery Cabbages
	Lettuce
	Celery
	Carrots
	Chinese Radish
	Sweet Potatoes
	Potatoes
	Pumpkins
	Lotus Roots
	Water Chestnuts
	Bamboo Shoots
	Hairy Melons
	Cucumbers
	Bitter Melons
	Winter Melons
	Tomatoes
	Red Capsicum/pepper
	Green Capsicum

	Sweet Corns
	Canned Corns
	Angled Loofah
	Eggplants
	Mungbean Sprouts
	Soybean Sprouts
10. Prebiotic vegetables	Asparagus
	Onions
	Chinese Chives
11. Meat	Pork, Lean
	Spare Ribs, Lean & Fat
	Spare Ribs, Lean
	Pork, Lean & Fat
	Pork Chop
	Beef Flank
	Beef Sirloin
	Briskets
	Lamb
12. Poultry	Chicken, with skin
	Chicken, without skin
	Chicken Strips
	Chicken wing, mid section
	Chicken Thigh
	Goose, with skin
	Goose, no skin
	Duck, with skin
	Duck, no skin
	Baby Pigeons
13. Fish	Grass Fish
	Big Head Fish
	Mud Carp
	Eel
	Japanese eel
	Blace
	Golden Thread/Horse Head
	Kwai Fa Fish
	Snake Head

	Carp
	Catfish
	Grouper
	Mackerel
	Ribbon Fish
	Big Eye Fish
	Canned Sardines
	Tuna Fish
	Salmons
14. Other seafood and shellfish	Squid
	Oysters
	Prawns
	Crabs
	Scallops/Dried Scallops
	Sea Cucumbers
	Cuttlefish
	Jelly Fish
15. Processed or marinated food	BBQ Pork, Lean & Fat
	Roast Pork, Lean
	Roast Pork, Lean & Fat (with 24% fat)
	Hot Dogs
	Big Red Sausage
	Chinese Sausage
	Chinese liver sausage
	Pork, Dried, Preserved & Salted
	Ham
	Canned luncheon Meat
	Hamburger Patties
	Fish Balls
	Fish Cakes
	Mud Carp Fish Balls
16. Animal livers	Pig Liver
	Chicken Liver
17. Eggs	Hard Boiled Eggs
	Quail Eggs
18. Whole milk	Whole Milk
	Chocolate Milk

	Whole Milk Powder
19. Low fat milk	Low fat milk
20. Skimmed milk	Skimmed Milk
	Skim Milk Powder
21. Dairy products (yogurt)	Whole Fat Yogurt
	Low Fat Yogurt
22. Dairy products (cheese)	Cheese
23. Dairy products (ice-cream)	Ice Cream
	Ice Cream Cone
	Ice cream bar
24. Other plant-based milk	Other plant-based milk
25. Soy milk	Soy Milk
	Vitasoy
26. Soy and soy products	Tofu
	Dried Tofu Sheets
	Deep Fried Tofu
	Deep Fried Tofu Pockets
	Tofu Skin
	Vegetarian Chicken
	Baked Beans
	Fresh Soybeans
	Tofu Fa
27. Other legumes	Red Bean
	Black Eye Peas
	Snap Beans
	Snow Peas
	Green Peas
	Broad Beans
	String Beans
28. Fungi and seaweeds	Fresh Mushrooms
	Dried Mushrooms
	Canned Mushroom
	Wood Fungus
	Black Moss
29. Nuts and seeds	Chestnut
	Cashew Nuts
	Peanut

30. Sugary snacks (desserts)	Milk Pudding
	Chinese Sweet Soup Desserts
31. Sugary snacks (candies)	Milk Chocolate
	Candies
32. Sugary snacks (cakes)	Spongy Cake
	Pound Cake
33. Sugary snacks (biscuits)	Egg Tart
	Semi-sweet Biscuits
	Chocolate Coated Biscuits
	Walnut Short Cakes
34. Soft drinks or sweetened beverages	Coca Cola
	Fanta Orange
	Sprite
	Yakult
	Canned/Bottled Juices
35. Diet or low sugar drinks	Diet coke
36. Sweetened tea or coffee	Coffee
37. Unsweetened tea	Chinese Tea
	Green Tea
	English Tea
38. Unsweetened coffee	Unsweetened coffee
39. Water	Plain Water
	Mineral Water
40. Fried food (dishes)	Chicken Nuggets
41. Fried food (snacks)	French Fries
	Potato Chips
	Hash Brown
42. Dim sum (dumplings)	Steamed Dim Sum
	Wonton
43. Dim sum (meat buns)	BBQ Pork Bun
44. Dim sum (sweet buns)	Sweet Bun w/ Lotus Seed Paste/Egg Yolk Filling
45. Dim sum (Chinese cakes)	Chinese Turnip Cake
46. Dim sum (rice rolls)	Steamed Cheung Fan (Steamed Rice Rolls)
47. Dim sum (fried)	Deep Fried Dumplings
48. Dim sum (other savory)	Chicken Paw
	Spare Ribs, Lean & Fat
49. Taste preference	Taste preference

50. Oils	Oils

Original FFQ food items that are not included in the shortened FFQ:

- Wheat Gluten
- Preserved Radish
- Lemons
- Fruit Cocktail in Syrup
- Chicken Heart
- Pig Heart
- Pig Kidneys
- Beef Offal
- Dried Oysters
- Fried Dace with Black Bean Sauce
- Salted Preserved Fish
- Century Eggs
- Salted Duck Eggs
- Condensed Milk
- Evaporated Milk
- Milk Shake
- Mayonnaise
- Chocolate Power Drink
- Horlick
- Ovaltine
- Fresh Fruit Juices
- Wine
- Spirits
- Light Beer
- Beer
- "Yau-Char-Kwai" (Deep Fried Dough)
- Pizza
- Hamburger
- Fish Burger
- Pork Pie
- Apple Pie
- Glazed Beef Jerky
- Glazed Pork Jerky
- Beef Floss
- Pork Floss
- Dried Squid Strings

- Saltines/Cream Crackers
- Honey
- Jam
- Peanut Butter
- Corn Syrup
- Herbs & Lean Pork Soup
- Carrots & Green Turnips Soup
- Watercress Soup
- Radish & Mud Carp Dace Soup
- Cabbages & Potatoes Soup
- Peanuts Chicken Paws Soup
- Split Peas & Peanut Soup
- Hairy Melons & Octopus Soup
- Cream of Chicken Soup
- Vegetable & Tofu Soup

Abbreviation: food frequency questionnaire (FFQ).
